# The State of the Art on Management of Patients with Unresectable Liver Metastases from Colorectal Cancer

**DOI:** 10.3390/biomedicines14071527

**Published:** 2026-07-07

**Authors:** Martim Porto, Beatriz Luciano, João Simões, Mónica Laureano, Inês Gil, Sara Pinheiro, Rui Caetano-Oliveira, Ricardo Martins, Miguel Coelho

**Affiliations:** 1 General Surgery Department, Unidade Local de Saúde da Região de Leiria, 2410-197 Leiria, Portugal; 2 Faculty of Medicine, University of Coimbra, 3004-531 Coimbra, Portugal; 3 Oncology Department, Unidade Local de Saúde da Região de Leiria, 2410-197 Leiria, Portugal; 4 Pathology Department of Germano de Sousa, 3000-377 Coimbra, Portugal

**Keywords:** colorectal liver metastases, unresectable disease, systemic chemotherapy, hepatic arterial infusion, transarterial chemoembolization, radioembolization, liver transplantation, conversion therapy

## Abstract

Colorectal cancer frequently metastasizes to the liver, and a substantial proportion of patients present with unresectable colorectal liver metastases (CRLM), which are associated with limited survival. While systemic chemotherapy remains a central component of management, advances in liver-directed therapies and transplantation have significantly expanded therapeutic possibilities in selected patients. This review provides a comprehensive and up-to-date overview of current management strategies for unresectable CRLM, with a focus on systemic chemotherapy, intra-arterial therapies, and liver transplantation. Systemic chemotherapy plays a central role, either as conversion therapy aimed at achieving secondary resectability or as palliative treatment to prolong survival and maintain quality of life. The integration of targeted agents and molecular profiling has enabled increasingly personalized therapeutic strategies. Liver-directed therapies, including hepatic arterial infusion chemotherapy, transarterial chemoembolization, and radioembolization, provide effective local disease control and may facilitate downstaging in selected patients. In parallel, liver transplantation has re-emerged as a promising option for highly selected patients with liver-only disease, demonstrating encouraging long-term survival in recent prospective studies. However, optimal patient selection, timing, and sequencing of these modalities remain key challenges. The management of unresectable CRLM is evolving toward a multidisciplinary and individualized approach that integrates systemic, locoregional, and transplant-based strategies. In selected patients, this paradigm shift may translate into meaningful survival benefit, although further prospective studies are required to refine indications and optimize treatment sequencing.

## 1. Introduction

Colorectal cancer (CRC) is the third most diagnosed malignancy and the second leading cause of cancer-related death worldwide [1]. A substantial proportion of CRC mortality is attributable to metastatic disease, with more than half of patients developing metastases during the course of their illness [2,3]. The liver and lungs are the most frequent metastatic sites, with metastases presenting either synchronously (at diagnosis or within 6 months) or as metachronous (more than 6 months after diagnosis of the primary tumor) [3,4].

In selected cases, metastatic CRC (mCRC) can be managed with curative intent through complete resection of both the primary tumor and metastases, combined with systemic therapy [5,6]. When an R0 resection is achieved (negative margins >1 mm in hepatic resection), the estimated 5-year overall survival rate ranges from 20% to 45% [2,4]. However, only about 20% of patients with colorectal liver metastases (CRLM) present with resectable disease at diagnosis [2,3,7,8].

Criteria for resectability have evolved considerably over time [9], and resectability remains partly institution-dependent, as surgical expertise and available liver-directed strategies may influence treatment eligibility. Accordingly, assessment should ideally be performed within a multidisciplinary team at experienced hepatobiliary centers. In general, resectability depends on the feasibility of complete tumor clearance while preserving an adequate functional future liver remnant (FLR), with acceptable vascular inflow/outflow and biliary drainage [2,3,6,9]. A subset of patients may fulfill technical resectability criteria but remain unsuitable for surgery because of comorbidities, poor performance status, or patient preference [4]. Management of unresectable CRLM requires multidisciplinary decision-making involving hepatobiliary surgeons, medical oncologists, radiologists, and interventional radiologists. Contemporary guidelines emphasize that treatment selection should integrate technical resectability, tumor biology, patient fitness, comorbidities, and treatment goals, with regular reassessment to identify opportunities for conversion to curative-intent strategies [4,10].

In recent years, it has become evident that strategies aiming to convert unresectable disease into resectable disease—known as conversion, downsizing or downstaging approaches—offer a survival advantage over palliative chemotherapy alone [9]. These strategies can be categorized according to the underlying cause of unresectability: (1) inability to achieve an R0 resection while preserving adequate hepatic function; (2) insufficient FLR; or (3) ineligibility for surgery.

For each scenario, different therapeutic approaches have been developed:

1. Technical-anatomical unresectability: systemic conversion chemotherapy, locoregional therapies such as transarterial radioembolization (TARE), transarterial chemoembolization (TACE) or hepatic arterial infusion chemotherapy (HAIC), and liver transplantation.
2. Insufficient FLR: portal vein embolization (PVE) or portal vein ligation (PVL) to induce hypertrophy of the FLR, associating liver partition and portal vein ligation for staged hepatectomy (ALPPS), or liver transplantation.
3. Patients inoperable or refusing surgery: systemic therapy alone, or minimally invasive locoregional or ablative techniques including TARE, TACE, radiofrequency ablation (RFA), or microwave ablation (MWA) [9].

When none of these strategies enables resection, systemic chemotherapy remains the only therapeutic option, albeit with a poor prognosis. The 5-year overall survival rate with palliative chemotherapy is approximately 11%, compared with 50–60% in patients undergoing combined treatment strategies [3].

**Figure 1:**
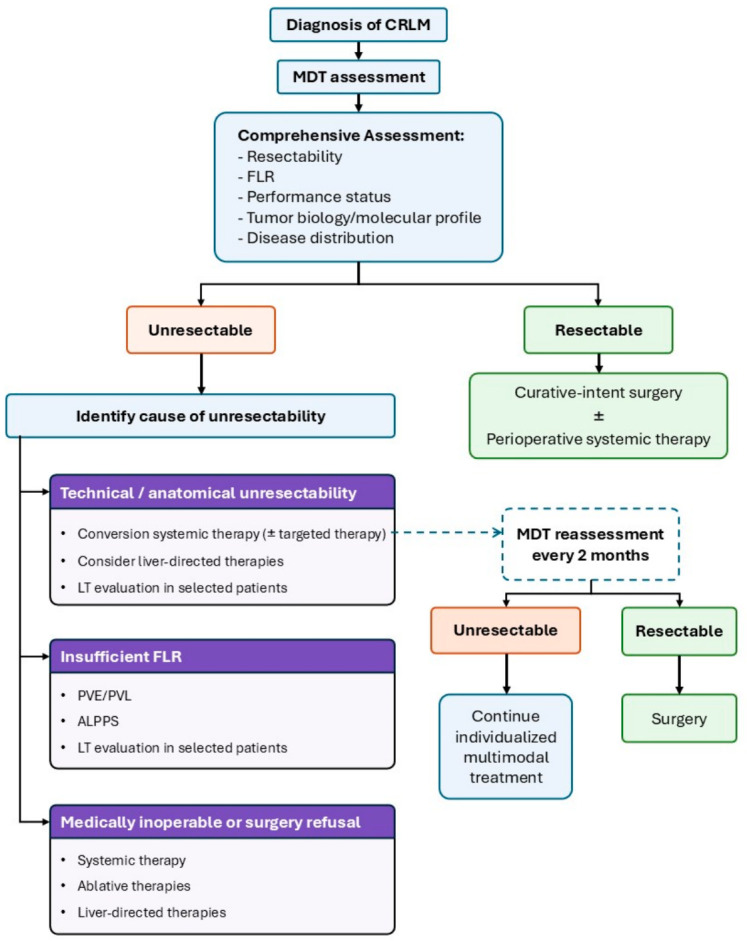
Conceptual framework for the management of unresectable colorectal liver metastases. This conceptual framework summarizes the major therapeutic pathways for patients with unresectable colorectal liver metastases according to the underlying cause of unresectability. It was developed as a schematic overview of the concepts discussed throughout this review and reflects contemporary evidence and recommendations from ESMO, ASCO, and NCCN guidelines [4,7,10]. It is intended to provide an overview of current management strategies rather than a prescriptive clinical algorithm. Final treatment decisions should be individualized within a multidisciplinary team according to tumor biology, disease distribution, patient fitness, treatment goals, and institutional expertise. CRLM, colorectal liver metastases; MDT, multidisciplinary team; FLR, future liver remnant; PVE, portal vein embolization; PVL, portal vein ligation; ALPPS, associating liver partition and portal vein ligation for staged hepatectomy; LT, liver transplantation.

The aim of this article is to provide a critical review of updated evidence regarding therapeutic options for patients with unresectable CRLM, with a particular focus on three main pillars: systemic chemotherapy, intra-arterial chemotherapy, and liver transplantation. A conceptual framework summarizing the major therapeutic pathways and decision domains in the management of unresectable CRLM is presented in Figure 1.

## 2. Systemic Chemotherapy

In metastatic colorectal cancer confined to the liver, the therapeutic strategy with the greatest curative potential—yielding a five-year overall survival rate of up to ~40%—is R0 surgical resection combined with systemic chemotherapy [6]. However, as previously noted, only a minority of patients are candidates for upfront resection at diagnosis [2]. Consequently, systemic therapy plays a central role in the management of most patients with CRLM, either to achieve secondary resectability or to control disease progression.

### 2.1. Therapeutic Goals: Conversion Versus Disease Control

In the setting of unresectable CRLM, chemotherapy can serve two distinct purposes: conversion or palliation [11]. Conversion chemotherapy (also referred to as OncoSurge) aims to render metastases resectable through downsizing and, once resected, to pursue curative intent [11]. Conversely, when inadequate response to systemic chemotherapy makes resection unlikely, curative treatment is deemed unattainable and the instituted therapy is termed palliative chemotherapy, with the goals of delaying progression, prolonging survival, and alleviating symptoms [4].

### 2.2. Molecular Profiling and Treatment Selection

Regimen selection depends on multiple factors, including therapeutic intent (conversion vs. palliation), primary tumor location, tumor histology and molecular biology, patient performance status and comorbidities, and the toxicity profile of the drugs, among others [4].

With respect to primary tumor location, right-sided colon cancers (proximal to the splenic flexure) display molecular features distinct from left-sided tumors [4]. Right-sided tumors more often exhibit mismatch-repair deficiency (dMMR) with high microsatellite instability (MSI-H), are more frequently mucinous, and have a higher prevalence of *RAS* and *BRAF* mutations [2,4]. Moreover, irrespective of treatment, right-sided tumors carry a worse prognosis [4]. By contrast, left-sided tumors tend to show chromosomal instability, EGFR (Epidermal Growth Factor Receptor) and HER2 (Human Epidermal Growth Factor Receptor 2) amplification, and aberrant EGFR signaling [2,4].

In recent years, there has been a shift toward increasingly personalized medicine, with current practice recommending genetic and molecular tumor profiling at diagnosis to guide chemotherapy selection according to disease characteristics [4,10]. Alterations with therapeutic impact include *RAS* and *BRAF* mutations, HER2 amplification and MSI status. These biomarkers help the choice of cytotoxic regimens, EGFR- or VEGF (Vascular Endothelial Growth Factor)-directed biologics, and immunotherapy [4,10].

Currently, before initiating any therapy, it is essential to assess the tumor molecular profile, including mutations in the *MAPK/ERK* pathway, as mutations in the *RAS* gene predict a lack of response to anti-EGFR monoclonal antibodies (mAbs) [2,4]. *RAS* mutations occur in ~25–52% of CRC and are more frequent in right-sided tumors (2). In addition, the *BRAF* V600E mutation is considered a poor prognostic factor in CRC [4,10]. In selected patients with *BRAF* V600E-mutant metastatic CRC and disease progression after first-line therapy, the phase III BEACON CRC trial demonstrated improved response rates, progression-free survival, and overall survival with encorafenib plus cetuximab compared with standard irinotecan-based therapy, establishing this combination as a standard treatment option in the refractory setting [12]. More recently, the phase III BREAKWATER trial further supported the role of targeted therapy in *BRAF* V600E-mutant metastatic CRC. In previously untreated patients, first-line treatment with encorafenib plus cetuximab and mFOLFOX6 significantly improved progression-free survival (12.8 vs. 7.1 months) and overall survival (30.3 vs. 15.1 months) compared with standard chemotherapy-based approaches, supporting earlier integration of targeted therapy in this molecular subgroup [13]. Cetuximab and panitumumab (anti-EGFR mAb) should be restricted to *RAS*-wild-type (*RAS*-wt) tumors [2,4].

However, in addition to the previously described alterations in the *MAPK/ERK* pathway, it is also important to consider that *HER2* gene amplification may be present in about 2–3% of patients with metastatic CRC [2]. HER2 amplification, in addition to being a poor prognostic factor, also appears to induce resistance to anti-EGFR mAbs through activation of alternative signaling pathways [2]. Therefore, patients with *RAS/BRAF* wild-type tumors should also be tested for HER2 amplification before initiating treatment with cetuximab or panitumumab, and may currently be candidates for anti-HER2 therapies, such as trastuzumab, in specific clinical contexts after progression on first-line systemic chemotherapy [2,3,10].

Microsatellite instability, in addition to being present in Lynch syndrome, can be identified in approximately 15–20% of sporadic colorectal cancers (CRC) and in 4–8% of metastatic CRC cases [2,7]. dMMR/MSI-H tumors, despite being poorly differentiated, show high response rates and durable responses, with a significant improvement in overall survival when treated with immune checkpoint inhibitors compared with cytotoxic chemotherapy regimens [2,4]. Following studies such as KEYNOTE-177 [14] and CheckMate 8HW [15], pembrolizumab (anti–PD-1 mAb) and the combination of nivolumab (anti–PD-1 mAb) plus ipilimumab (anti–CTLA-4 mAb) have become considered first-line therapeutic options for unresectable metastatic CRC with dMMR/MSI-H [3,8,10].

Another therapeutically actionable biomarker in later-line settings is NTRK fusions [4]. Owing to their low incidence (<0.5%), testing is not advised upfront but after failure of standard therapy; if detected on broad genomic profiling, larotrectinib or entrectinib can be considered among the therapeutic options [4]. NTRK fusions are more frequent in right-sided colon tumors [4].

The investigation of other biomarkers—such as *ALK* and *ROS1* fusions and *PIK3CA* mutations—is not yet recommended routinely, although used in some clinical trials [4].

Given the centrality of fluoropyrimidines (5-fluorouracil (5-FU) or capecitabine) in metastatic CRC regimens, EMA (European Medicines Agency) and ESMO recommend testing for *DPYD* gene polymorphisms, which can cause dihydropyrimidine dehydrogenase (DPD) deficiency, a key enzyme in fluoropyrimidine metabolism [2,4]. DPD expression influences chemosensitivity and the risk of adverse reactions to fluoropyrimidines [2,4].

### 2.3. Conversion Therapy

The goal of conversion chemotherapy is achieving resectability, not complete pathologic response [11]. Recent meta-analyses indicate that contemporary regimens achieve conversion rates of 5–15%, with five-year overall survival rates of ~30–35% among patients who become resectable [8]. Compared with chemotherapy alone (20% five-year overall survival rate), outcomes are superior when conversion chemotherapy is followed by metastasectomy, even when disease was unresectable at presentation [8]. Nonetheless, despite high response rates, not all patients reach resectability [6]. Thus, systemic chemotherapy remains the principal approach in patients with permanently unresectable CRLM [7].

Although no single regimen is established as best, there is consensus across NCCN (National Comprehensive Cancer Network) [10], ASCO (American Society of Clinical Oncology) [7] and ESMO (European Society for Medical Oncology) [4] regarding the main first-line options for unresectable CRLM and their indications [3,4,7,8,10]. Current international guidelines converge on combining cytotoxic chemotherapy with targeted agents as one of the most effective strategies to convert initially unresectable disease to resectable status, particularly in liver-only involvement [3,11]. Doublet chemotherapy regimens such as FOLFOX and FOLFIRI (5-FU, leucovorin, and oxaliplatin or irinotecan, respectively) became standard in the 2000s [11]. Subsequently, the triplet FOLFOXIRI (5-FU, leucovorin, oxaliplatin, irinotecan) further improved response rates and the likelihood of curative resection in both first- and second-line settings [11]. Adding targeted therapy—anti-EGFR or anti-VEGF mAbs—to these backbones enhanced outcomes, with response rates >50% and median overall survival approaching 30 months [11]. Anti-EGFR therapy should be reserved for *RAS*-wt tumors, whereas anti-VEGF use is not molecularly restricted [11]. Key clinical trials supporting conversion therapy strategies are summarized in Table 1.

Among doublets, mFOLFOX6 plus bevacizumab (anti-VEGF mAb) stands out, achieving a 23.1% surgical conversion rate, including 15.4% R0 resections [3]. In TRICC0808 [16], patients undergoing hepatectomy after this regimen had a median overall survival of 43.1 months, although with high recurrence rate. Similar findings in phase II CELIM [17] and PLANET [18] showed prolonged survival rates in initially unresectable *RAS*-wt CRLM who responded to anti-EGFR-based conversion therapy and subsequently underwent hepatic resection [3]. These data reinforce the role of conversion therapies not only in inducing response but also in improving long-term outcomes in selected patients [3].

However, the use of targeted therapies in the perioperative setting for technically resectable colorectal liver metastases should be avoided [8]. The phase III New EPOC trial [19], which evaluated patients with resectable liver metastases, demonstrated unfavorable outcomes with the addition of cetuximab to chemotherapy, showing a significant reduction in overall survival in the cetuximab plus chemotherapy group (55.4 months) compared with the chemotherapy-alone group (81.0 months) [8]. These findings argue against the use of cetuximab in the perioperative setting of resectable disease and reinforce the need for caution in this context [8]. In contrast, in situations where the goal is conversion of initially unresectable or potentially resectable disease, the addition of cetuximab to doublet chemotherapy may be considered, given its efficacy in increasing tumor response and surgical conversion rates [8].

Among triplets, FOLFOXIRI ± bevacizumab is highly effective for conversion to hepatic resectability [8]. The phase III CAIRO5 trial [20], which enrolled 296 patients and included a pre-specified cohort of patients with right-sided and/or *RAS/BRAF*-mutant unresectable CRLM (*n* = 219), demonstrated that treatment with FOLFOXIRI plus bevacizumab for up to six months resulted in a significant improvement in progression-free survival (10.6 vs. 9.0 months) and a higher rate of R0/R1 resections (51% vs. 37%) compared with FOLFOX or FOLFIRI plus bevacizumab [8]. Trials such as TRIBE [21] and OLIVIA [22] also reported high hepatic resection rates with FOLFOXIRI ± bevacizumab, although with greater toxicity [3]. A meta-analysis by Tomasello et al. [23] (11 studies; 877 patients) found a 39% surgical conversion rate with FOLFOXIRI–bevacizumab, including 28.1% R0 resections [3].

Despite its benefits in terms of response rate and conversion to curative surgery, given its toxicity, FOLFOXIRI should be reserved for carefully selected patients; it is not recommended for those >75 years, with performance status ≥ 2, or with significant comorbidity. It is best suited to younger, fit patients for whom an intensive path-to-resectability strategy is intended [4].

These conversion and resection figures should be interpreted with caution rather than compared directly across studies. Reported conversion rates vary widely, from approximately 5–15% in unselected contemporary series to 23–51% in trial cohorts [8,16,17,18,20,22,23], largely reflecting differences in patient selection and in the definition of unresectability rather than regimen efficacy alone; notably, only CAIRO5 applied prospective central-panel adjudication of resectability [20], whereas most supporting data derive from phase II trials (CELIM, PLANET, OLIVIA, TRICC0808) [16,17,18,22] or pooled analyses of predominantly non-randomized studies [23]. Weighted by level of evidence, the strongest (phase III) data come from CAIRO5, in which intensification to FOLFOXIRI–bevacizumab produced only a modest progression-free survival gain (10.6 vs. 9.0 months) at the cost of greater toxicity, with the survival advantage driven chiefly by achieving secondary resection across arms; in the left-sided *RAS/BRAF* wild-type cohort, the addition of panitumumab to a doublet did not improve progression-free survival over bevacizumab (10.4 vs. 10.8 months) and increased toxicity [20]. Conversely, New EPOC (phase III) showed that anti-EGFR therapy is not merely ineffective but harmful in the upfront-resectable setting, where adding cetuximab significantly shortened overall survival (55.4 vs. 81.0 months) [19]. Taken together, these data support FOLFOXIRI–bevacizumab as the best-evidenced option for fit patients with adverse tumour biology, while underscoring that cross-study conversion rates are hypothesis-generating and that regimen choice must remain biology- and setting-specific.

**Table 1:** Major clinical trials evaluating conversion therapy strategies in initially unresectable colorectal liver metastases.

Trial	Population	Regimen	Conversion/Resection Outcomes	Key Outcomes	Study Design (Level of Evidence)
TRICC0808 [16]	Liver-only unresectable CRLM	mFOLFOX6 + bevacizumab	R0 resection: 44.4%	Median OS 2.8 years overall; 3-year OS 61.3% after resection	Phase II (IIb)
CELIM [17]	Unresectable CRLM	Cetuximab + FOLFOX6/FOLFIRI	Resectability increased from 32% to 60%; R0 resection 34%	Response rates 57–68%	Randomized phase II (IIb)
PLANET [18]	WT-*KRAS* unresectable liver-limited disease	Panitumumab + FOLFOX4/FOLFIRI	R0/R1 resection 34–46%	Median OS 37–41 months	Phase II (IIb)
OLIVIA [22]	Unresectable liver-only CRLM	FOLFOXIRI + bevacizumab	R0/R1 resection 51.2%	Median PFS 18.6 months	Randomized phase II (IIb)
CAIRO5 [20]	Initially unresectable CRLM with right-sided and/or *RAS/BRAF*-mutant tumors	FOLFOXIRI + bevacizumab	R0/R1 resection ± ablation 51.4%	Median PFS 10.6 months	Phase III RCT (Ib)
Tomasello et al. [23]	Meta-analysis (877 patients)	FOLFOXIRI + bevacizumab	Conversion 39%; R0 resection 28.1%	Pooled evidence supporting conversion strategy	Pooled meta-analysis (IIa)

CRLM, colorectal liver metastases; OS, overall survival; PFS, progression-free survival; RCT, randomized controlled trial.

Successful conversion depends not only on choosing the most effective first-line regimen but also on appropriate duration [11]. It is important to stress that the aim of conversion chemotherapy is resectability—not complete pathologic response [11]. Prolonging treatment beyond what is necessary risks cumulative toxicity, vanishing liver metastases (hindering adequate resection), and even tumor progression that may forfeit the surgical window [3,11].

Regarding chemotherapy-induced liver injury, irinotecan-containing regimens are linked to steatohepatitis (*yellow liver*), while oxaliplatin is associated with sinusoidal obstruction syndrome (*blue liver*) [3,8,10,11]. These histologic changes carry clinical consequences, with a 20–25% increase in postoperative complications versus surgery alone [3]. To mitigate risk, patients on neoadjuvant conversion therapy should undergo frequent reassessment, ideally every two months [8]. Some analytic studies have shown that gamma-glutamyl transferase (GGT) levels may have a role in predicting sinusoidal obstruction syndrome lesions [24]. Once resectability is achieved, chemotherapy should be stopped, and surgery scheduled after an appropriate interval: 3–4 weeks for chemotherapy alone or combined with anti-EGFR mAbs, and 5–6 weeks if bevacizumab was administered in the last cycle [4,10]. Bevacizumab may exert a protective effect against oxaliplatin-induced sinusoidal obstruction syndrome without worsening postoperative morbidity or mortality [3]. Overall, the total duration of preoperative conversion chemotherapy should not exceed four months [10].

When synchronous CRLM become resectable, patients should undergo synchronous or two-stage resection of the primary tumor and liver metastases, as no strategy has been shown to be superior [10]. In contrast, with permanently unresectable CRLM, the current literature discourages initial resection of an asymptomatic primary tumor [4]. Palliative resection of the primary tumor should be reserved for obstruction, significant bleeding, or perforation [10].

### 2.4. Management of Permanently Unresectable Disease

Major guidelines (NCCN, ASCO, ESMO) primarily emphasize conversion strategies, with comparatively sparse guidance for disease that is clearly unresectable at baseline or remains unresectable after treatment. This gap underscores the need for a truly patient-centered approach, tailored to clinical frailty and realistic therapeutic potential, as well as the importance of a multidisciplinary approach in referral centers [4,7,10].

In this setting, systemic chemotherapy is the standard of care, primarily to control symptoms, preserve quality of life, and prolong survival [6]. Decisions should be individualized, incorporating age, performance status, comorbidities, tumor location and biology, and patient preferences [4]. In frail patients or those with relevant comorbidities who cannot tolerate aggressive combinations, less toxic options are preferable: fluoropyrimidine monotherapy ± bevacizumab, or anti-EGFR monotherapy in *RAS*-wt tumors [4]. In these cases, a sequential strategy—the so-called *continuum of care*—can maximize benefit over time despite the absence of curative options. Debulking surgery or ablation without curative intent are not recommended in these patients [10].

### 2.5. Limitations and Future Perspectives

Before addressing emerging tools and future directions in the management of CRLM, it is important to establish a clear distinction between two categories of biomarkers and technologies: (1) those currently integrated into routine clinical practice and supported by prospective evidence sufficient to guide treatment decisions; and (2) those that, while scientifically promising, remain investigational, not yet suitable for routine clinical use, pending prospective interventional validation, methodological standardization, and demonstration of reproducibility across institutions. Biomarkers currently validated and recommended for routine molecular profiling, including *RAS*, *BRAF* V600E, HER2 amplification, MSI/dMMR status, and *DPYD* polymorphisms, have been discussed in Section 2.2. The remainder of this section addresses the second category: emerging tools whose translational potential is recognized but whose clinical implementation remains premature.

Despite the advances described in the preceding sections, meaningful limitations in systemic therapy remain: (1) modest conversion rates (~5–15%), (2) secondary resistance, and (3) toxicity that limits intensified regimens like FOLFOXIRI to younger, fit patients [3,4,8]. Even after resection, recurrence rates remain high, reflecting the systemic nature of the disease [3]. The paucity of guidance for disease that is unresectable at baseline or remains unresectable after multiple lines further reinforces the need for individualized, patient-centered decisions [4].

It is in this context that emerging investigational tools are being developed to better characterize tumor biology and refine treatment selection. Among these, the histological growth patterns (HGP) of colorectal cancer metastases have provided new insights into tumor biology [25]. HGP assessment has demonstrated reproducibility and prognostic implications [26], with several studies confirming its impact on outcomes in CRLM [27,28] and in metastases from other primary tumors [29,30]. New discoveries have deepened this knowledge, characterizing the biological meaning of the desmoplastic/encapsulated and non-desmoplastic profiles [31]: the desmoplastic pattern exhibits predominantly angiogenic features, while the non-desmoplastic pattern relies on vessel co-option mechanisms for growth [31,32,33]. These biological distinctions have generated the hypothesis that HGP-guided treatment selection, specifically the preferential use of anti-angiogenic agents in patients with desmoplastic CRLM, could improve therapeutic outcomes. However, this hypothesis has not yet been tested in prospective interventional trials, and its clinical translation therefore remains unestablished. Furthermore, HGP assessment currently requires resected hepatic tissue, limiting its applicability to patients who have already undergone surgery [33,34]. While some imaging studies have demonstrated exploratory predictive value for preoperative HGP estimation [35,36], and certain primary tumor features have been investigated as potential surrogates [37,38], these approaches remain preliminary. Genetics do not appear to play a determining role in HGP phenotype [39], with the phenomenon likely being more dependent on the tumor microenvironment [40]. Emerging radiomics and artificial intelligence-based imaging approaches may further improve non-invasive HGP prediction in the future [35,36]; however, significant barriers remain, including limited reproducibility across imaging protocols, absence of standardized methodologies, and lack of prospective validation in interventional settings. Until these barriers are addressed, HGP should be regarded as a research tool with recognized prognostic interest rather than an instrument for routine clinical decision-making [34,35,36].

Looking ahead, practice is moving toward more personalized and adaptive oncology, integrating “non-classical” biomarkers (e.g., HER2, *POLE/POLD1*, *NTRK* fusions) to broaden therapeutic options beyond established regimens [2,4]. Liquid biopsy, particularly circulating tumor DNA (ctDNA), represents a promising investigational tool in this context. Retrospective and exploratory data suggest potential applications in risk stratification, minimal residual disease detection, and recurrence surveillance in patients with CRLM [41,42]; however, its role in prospective treatment selection and real-time therapeutic adaptation has not yet been established in interventional trials, and ctDNA-based decision-making should not be considered part of standard clinical assessment at the present time. Additional precision oncology approaches, including antibody-drug conjugates, are currently under investigation and may further expand treatment options in selected molecular subgroups [43]. Beyond the boundaries of systemic therapy, and in the broader context of translational oncology research, emerging multimodality imaging strategies are also being explored for potential applications in oncological imaging and treatment response assessment, including hybrid nuclear medicine approaches that combine radioactive and non-radioactive molecular probes for enhanced tumor characterization [44] and optical molecular imaging techniques targeting tumor-specific antigens such as PD-L1 in colorectal cancer models [45]. These approaches are currently preclinical or at an early conceptual stage and have not been evaluated in the clinical management of CRLM; their potential relevance to future treatment personalization strategies warrants acknowledgment in the context of this evolving landscape.

At the level of therapeutic strategy, selective intensification (for conversion) and de-intensification (for frail patients), guided by molecular profiles and clinical performance, aim to optimize the efficacy-toxicity balance [3,4]. Ongoing trials, namely COLT (NCT03803436), SACA III (NCT03494946), and SOULMATE (NCT04161092), should clarify the relative roles of intensified chemotherapy, surgery, and liver transplantation, the latter already supported by positive TransMet data [6]. Finally, rational integration of locoregional therapies (e.g., SIRT, intra-arterial chemotherapy) with systemic regimens may improve hepatic control in liver-only disease, although robust evidence is still needed to define the subgroups that benefit most [3,8].

## 3. Local Therapies for Unresectable CRLM

Surgical metastasectomy offers the highest chance of cure, yet most patients are not candidates for resection at diagnosis [2,46,47]. For those unsuitable for surgery due to tumor distribution, comorbidities, insufficient hepatic reserve, or personal refusal, various liver-directed local therapies are available [2,46,48].

Local treatments are particularly relevant in the context of oligometastatic disease and are broadly classified into ablative and intra-arterial techniques, depending on their method and therapeutic intent [2,47,48]. Ablative therapies, such as radiofrequency ablation (RFA) and microwave ablation (MWA), are thermal modalities that may provide durable local control and long-term survival in carefully selected non-surgical candidates [2,47,48,49]. Stereotactic body radiation therapy (SBRT) represents a non-thermal alternative [2,47,48]. Intra-arterial approaches such as hepatic arterial infusion chemotherapy (HAIC), transarterial chemoembolization (TACE) and transarterial radioembolization (TARE) exploit the liver’s dual blood supply, targeting tumors through the hepatic artery while sparing normal parenchyma [2,46,47,48]. A comparative summary of the principal locoregional treatment modalities, including indications, ideal clinical settings, limitations, and levels of evidence, is provided in Table 2.

These therapies play a vital role in disease control and may delay or even eliminate the need for systemic treatment, particularly in patients with indolent tumor biology [2,47,48,50]. Selection of local treatment is influenced by lesion size, anatomical location, and *RAS* mutation status [2,48,49].

1. Ablative therapies
  a. Radiofrequency ablation

Radiofrequency ablation (RFA) is a minimally invasive thermal technique that induces coagulative necrosis in hepatic tumors and adjacent parenchyma [51,52,53]. It demonstrates the highest efficacy in patients with solitary or few metastases smaller than 3 cm [51,52,53,54,55]. However, its effectiveness may be limited in lesions adjacent to large blood vessels due to the heat-sink effect and should be cautiously applied to tumors near the hepatic dome or inferior edge to prevent diaphragmatic or bowel injury [51,52,53,56]. Techniques such as hydrodissection may help mitigate thermal damage to nearby structures [51,53]. 

RFA provides promising local tumor control and survival outcomes in patients with solitary metastases, although data on colorectal liver metastases remain heterogeneous, with five-year survival rates ranging from 14% to 55% and local recurrence rates between 3.6% and 60% [51,52,53,54,55,57,58,59,60,61]. The current evidence is limited and often includes mixed populations, encompassing both resectable and unresectable liver disease, with or without extrahepatic spread [51,52,53,54,55]. 

While generally well tolerated, RFA may be associated with serious complications. Postablation syndrome, affecting 30–40% of patients, is the most common adverse effect, typically self-limiting within ten days [52,53]. Follow-up imaging with contrast-enhanced CT or MRI is performed from one month post-treatment, with necrotic lesions typically appearing hypodense and often larger than the original tumor [60,61]. Given its repeatability, low complication rate, and minimal disruption to systemic therapy, RFA is gaining recognition as a potential alternative to surgical resection in selected patients [51,53,54,55]. 

Recent evidence from the phase III COLLISION trial has strengthened the role of thermal ablation in the management of small (≤3 cm) colorectal liver metastases, demonstrating non-inferiority to surgical resection regarding overall survival, with reduced treatment-related morbidity [62].

  b. Microwave ablation

Microwave ablation (MWA) is a percutaneous locoregional therapy that resorts to electromagnetic energy to induce thermal coagulative necrosis through molecular friction [63,64]. It has seen increasing adoption in the management of liver metastases due to several technical advantages over other ablative modalities, including shorter procedure times, reduced procedural discomfort, the ability to treat larger tumors, and lower susceptibility to the heat sink effect caused by adjacent blood vessels [63,64,65]. 

MWA is indicated for patients deemed unfit for surgery or with unresectable hepatic lesions [63,64,66,67]. Compared to radiofrequency ablation (RFA), MWA can treat larger and more centrally located tumors [63,64,65]. Reported five-year overall survival (OS) rates reach approximately 37%, which is considered favorable in this patient population [63,68,69,70]. Although MWA and RFA share a similar side effect profile, complications associated with MWA are generally milder [63,64,65]. 

While no randomized controlled trials directly comparing RFA and MWA are currently available, clinical practice tends to favour MWA for lesions adjacent to large vessels due to its reduced sensitivity to heat sink effects [63,64,65,71]. Conversely, RFA remains the preferred option for peribiliary lesions [63,64,65]. 

The growing role of MWA is further supported by recent evidence from the phase III COLLISION trial, which demonstrated that thermal ablation is non-inferior to surgical resection for small (≤3 cm) resectable colorectal liver metastases, while being associated with lower morbidity and shorter hospital stay [62].

  c. Stereotactic Body Radiation Therapy (SBRT)

Stereotactic body radiotherapy (SBRT) is a highly conformal external beam radiotherapy technique that delivers ablative doses of radiation to liver metastases using advanced 4D imaging for tumor tracking [72,73,74,75]. This approach enables precise targeting of lesions while sparing adjacent healthy liver tissue and critical structures, making it particularly suitable for patients with limited disease burden or those unfit for surgery [72,73,74,75]. 

Clinical data, derived predominantly from retrospective and prospective non-randomized studies, suggest that SBRT may provide favorable local control with an acceptable toxicity profile in selected patients [72,75,76,77,78,79,80]. Most studies have treated between one and three hepatic lesions, although sequential SBRT has been successfully employed for patients with multiple metastases, with reported five-year overall survival (OS) rates reaching 57% in selected cohorts [75,77,78,79]. In a meta-analysis of 18 non-randomized studies involving patients with one to five liver metastases (many of whom had previously received systemic chemotherapy), one- and two-year OS rates were 67% and 57%, respectively, with corresponding local control rates of 67% and 59% [80]. 

SBRT is particularly valuable in elderly or medically inoperable patients, and in those with oligometastatic disease amenable to local treatment [72,75,78]. Available comparative studies suggest that SBRT may achieve outcomes comparable to thermal ablation in selected clinical scenarios, although direct randomized comparisons remain limited [72,75]. Some retrospective analyses have reported superior local control with SBRT for lesions >2 cm compared with RFA, although these findings should be interpreted cautiously given potential selection bias, while for tumors ≤2 cm, RFA may be more effective [72,75]. 

SBRT may also be preferred for lesions adjacent to large blood vessels, where heat-sink effects can reduce the efficacy of thermal ablation [72,75]. Ultimately, the choice between SBRT and ablation is often guided by lesion characteristics, anatomical considerations, local expertise, and patient preference [72,75].

2. Intra-Arterial Therapies
  a. - Hepatic arterial infusional chemotherapy

Hepatic arterial infusion chemotherapy (HAIC) is a locoregional therapeutic strategy for the management of CRLM, based on the anatomical principle that hepatic tumors derive the majority of their blood supply from the hepatic artery, in contrast to normal liver parenchyma, which is predominantly perfused by the portal vein [81,82,83,84,85]. 

This technique enables the targeted delivery of chemotherapeutic agents—most commonly floxuridine (FUDR)—directly into the hepatic artery via a surgically or percutaneously implanted catheter, typically placed in the gastroduodenal artery. FUDR is favored due to its high hepatic extraction and short half-life, allowing intratumoural drug concentrations up to 400 times greater than those achievable with systemic administration, while minimizing systemic toxicity [81,84,85,86]. Although alternative agents such as oxaliplatin and irinotecan have been used, particularly in Europe and Asia, their pharmacokinetic profiles are generally less advantageous [83,87,88,89]. 

HAIC has shown promising activity as a downstaging strategy and may facilitate conversion of selected patients with initially unresectable CRLM to resectable disease [81,90]. Selected studies from specialized centers have reported objective response rates approaching 85% in previously treated patients and exceptionally high response rates in chemotherapy-naïve populations. Conversion-to-resection rates exceeding 50% have also been described. However, these results should be interpreted in the context of highly selected patient populations and may not be broadly generalizable [81,82,85,86,90]. Crucially, these rates are not directly comparable with the 5–15% conversion observed in unselected systemic-therapy populations [8]: they derive from single-centre cohorts without phase III validation [81,82,85,86,90], and the difference reflects patient selection as much as treatment effect.

  b. Transarterial Chemoembolization

Transarterial Chemoembolization (TACE) is a minimally invasive, image-guided locoregional therapy that combines targeted intra-arterial chemotherapy with embolization of the tumor’s arterial supply. Unlike thermal ablative techniques, TACE does not rely on direct heat-induced cytotoxicity but rather induces tumor necrosis through ischemia and high local concentrations of chemotherapeutic agents [91,92]. 

TACE is most established in the treatment of hepatocellular carcinoma but has also been explored in the management of unresectable colorectal liver metastases, particularly in patients who are refractory to systemic therapy or unsuitable for ablation or resection [91,92,93,94,95,96,97]. Reported response rates have been encouraging across several studies, with objective response rates ranging from 40–60% and disease control rates exceeding 80%; however, the available evidence is largely derived from heterogeneous cohorts and non-randomized studies with median overall survival ranging from 12 to 25 months depending on prior treatment and tumor burden [91,92,94,95,96]. 

TACE can serve as salvage treatment or bridge to other therapies and may be used alone or in combination with systemic chemotherapy [91,95,96,97]. A randomized DEBIRI trial reported improved overall survival (22 vs. 15 months) and progression-free survival (7 vs. 4 months) compared with systemic FOLFIRI [97]. Moreover, newer combinations with anti-angiogenic agents and immunotherapy are under investigation, aiming to potentiate TACE’s local effects with systemic disease control [93,94]. 

TACE is best suited for patients with liver-dominant disease, preserved liver function, and no significant extrahepatic progression. Complications include post-embolization syndrome (pain, fever, nausea), transient liver enzyme elevations, and, less commonly, biliary or vascular injury [91,92,95]. 

Although not considered curative, TACE may provide disease control and symptom palliation in carefully selected patients with colorectal liver metastases. Further prospective studies are needed to define its optimal timing, combination strategies, and long-term oncological impact [91,92,93,94,95,96,97].

  c. Transarterial radioembolization

Transarterial Radioembolization (TARE), also referred to as Selective Internal Radiation Therapy (SIRT), is a form of intra-arterial brachytherapy utilizing Yttrium-90-laden microspheres, either resin or glass, which are selectively administered via branches of the hepatic artery [98,99,100]. This technique enables the delivery of high-dose radiation to hypervascular liver metastases, with a limited tissue penetration of approximately 2.5 mm, thereby preserving surrounding healthy tissue [98,99]. 

SIRT is generally better tolerated than transarterial chemoembolization (TACE) [101,102,103,104,105,106]. In the context of unresectable metastatic colorectal cancer, SIRT has not demonstrated a survival benefit when combined with first-line systemic chemotherapy [99,100]. Moreover, a higher incidence of adverse effects has led to its exclusion from routine use in this setting, as per current expert guidelines [99,100]. However, some evidence suggests improved overall survival in patients with right-sided primary tumors compared to left-sided disease, though this has not altered standard recommendations [99,100]. 

In the second-line setting, SIRT has been associated with enhanced progression-free survival when used alongside systemic chemotherapy. This benefit was confirmed in the phase III EPOCH trial, where the addition of Yttrium-90 radioembolization to standard second-line chemotherapy significantly improved progression-free survival and hepatic progression-free survival in patients with liver-dominant metastatic colorectal cancer, although no statistically significant improvement in overall survival was demonstrated [100,102,107]. 

The principal indications, limitations, and evidence supporting currently available locoregional therapies are summarized in Table 2.

**Table 2:** Summary of the main locoregional treatment modalities for unresectable colorectal liver metastases.

Technique	Main Indications	Ideal Lesion Characteristics	Main Limitations and Contraindications	Level of Evidence
RFA [51,52,53,54,55,56,57,58,59,60,61]	Unresectable CRLM; patients unfit for surgery; selected resectable oligometastatic disease	≤3 lesions; peripheral location; away from major vessels and biliary structures	Heat-sink effect with reduced efficacy near large vessels; risk of diaphragmatic or bowel injury; higher local recurrence in larger lesions. *Relative contraindications:* lesions > 3 cm or multifocal disease beyond the oligometastatic range; perivascular location precluding adequate margins; lesions abutting diaphragm or bowel without protective measures	Ib (Phase III COLLISION supports thermal ablation for ≤ 3 cm lesions) [62]
MWA [63,64,65,66,67,68,69,70,71]	Same indications as RFA; increasingly preferred thermal ablation modality	≤3–5 cm; lesions near large vessels; centrally located tumors	Limited prospective comparison with RFA. *Relative contraindications:* peribiliary lesions (RFA preferred); significant extrahepatic disease	Ib (Thermal ablation supported by Phase III COLLISION trial) [62]
SBRT [72,73,74,75,76,77,78,79,80]	Medically inoperable patients; oligometastatic disease; lesions unsuitable for ablation	1–3 lesions; lesions adjacent to major vessels; tumors >2 cm	Mostly non-randomized evidence; risk of radiation-induced liver injury; requires specialized expertise. *Relative contraindications:* diffuse or extensive hepatic disease beyond the oligometastatic range; insufficient uninvolved liver volume/impaired hepatic reserve	IIb (Prospective non-randomized studies and meta-analyses) [77,79,80]
HAIC [81,82,83,84,85,86,87,88,89,90]	Liver-dominant unresectable CRLM; conversion-to-resection strategy	Multifocal liver-only disease; preserved liver function; limited extrahepatic disease	Technically demanding; catheter-related complications; available only in specialized centers. *Contraindications:* significant extrahepatic disease; impaired hepatic function; unfavourable hepatic arterial anatomy precluding catheter placement	IIb (Large institutional series; no phase III evidence) [85]
TACE [91,92,93,94,95,96]	Salvage treatment; liver-dominant unresectable disease refractory to systemic therapy	Hypervascular or liver-dominant disease; preserved liver function	Post-embolization syndrome; limited survival benefit; lack of robust randomized data; uncommon biliary or vascular injury. *Contraindications:* significant extrahepatic progression; impaired hepatic function/decompensated liver disease	IIb (Phase III DEBIRI trial [97] and retrospective studies [91,92,93,94,95,96])
SIRT/TARE [98,99,100,101,102,103,104,105,106,107]	Liver-dominant unresectable CRLM; selected second-line treatment; downstaging in selected cases	Predominantly hepatic disease; preserved liver function; limited extrahepatic progression	No OS benefit in first-line setting; radiation-related toxicity; costly and technically complex. *Contraindications:* significant hepatopulmonary (lung) shunting; inadequate hepatic reserve; significant extrahepatic disease; high risk of non-target deposition	Ib (Phase III EPOCH showed improved PFS but no OS benefit) [107]

CRLM, colorectal liver metastases; RFA, radiofrequency ablation; MWA, microwave ablation; SBRT, stereotactic body radiotherapy; HAIC, hepatic arterial infusion chemotherapy; TACE, transarterial chemoembolization; SIRT/TARE, selective internal radiation therapy/transarterial radioembolization; OS, overall survival; PFS, progression-free survival.

## 4. Liver Transplantation for Unresectable CRLM

Liver transplantation (LT) has re-emerged as a promising therapeutic strategy for a carefully selected subset of patients with unresectable CRLM [5,108,109,110]. While systemic chemotherapy continues to play an important role within the multidisciplinary management of this population—aiming to delay disease progression, improve quality of life, and potentially enable secondary resection—long-term survival remains limited [4,7,10,108,109,110]. Even with advances in systemic regimens, only 20–40% of patients become eligible for hepatic resection, and the five-year overall survival (OS) rate in unresectable cases has historically hovered around 10% [4,7,10].

Recent data from prospective studies and institutional experiences have challenged this paradigm [5,108,109,110]. The SECA trials from Oslo, Norway, have provided encouraging evidence suggesting a survival benefit of LT in highly selected patients with liver-only CRLM [5,108,109,110]. In SECA-II, five-year OS reached 83% in patients meeting stringent inclusion criteria, and subsequent analyses have confirmed that low metabolic tumor burden, CEA levels, and a disease-free interval of at least one year are important prognostic markers [5,108,109,110]. The Oslo score has become a widely referenced tool in assessing transplant eligibility, and ongoing refinements in patient selection continue to enhance outcomes [5,108,109,110].

The landmark TransMet randomized controlled trial further strengthened the evidence base by demonstrating significantly improved five-year OS in patients undergoing LT combined with chemotherapy (56.6%) compared to those receiving chemotherapy alone (12.6%). This has reinforced the notion that, under strict conditions, LT may substantially improve long-term survival in carefully selected patients who would otherwise have limited therapeutic options, although recurrence remains common and the curative potential of this approach remains uncertain [6,9,108,109,110]. Within this evidence base, TransMet provides the only level I (randomized) data [6], whereas the SECA experience and living-donor series remain non-randomized, single-centre cohorts [5,108,109,110]; the consistency of the survival signal across these designs is reassuring, but the strength of any recommendation should be calibrated accordingly.

Despite early setbacks in the 1980s and 1990s—marked by high postoperative mortality, frequent graft loss unrelated to tumor recurrence, and limited survival—recent advances in surgical technique, immunosuppression protocols, and perioperative care have improved both graft viability and oncologic outcomes [108,109,110]. Moreover, the experience accumulated through LT for hepatocellular carcinoma and cholangiocarcinoma has contributed to the development of more refined oncological transplant strategies [108,109,110].

Living donor liver transplantation (LDLT) has emerged as a feasible solution to the issue of organ scarcity and MELD-based allocation limitations [109,110]. Early experiences from specialized centers in North America and Europe have reported favorable short- and mid-term outcomes, although longer follow-up and external validation are still required, with some achieving one-year OS rates of 100% and three-year disease-free survival (DFS) exceeding 60% [109,110]. The use of marginal grafts supported by advances in machine perfusion technologies such as normothermic perfusion further expands the donor pool and offers additional avenues for safe transplantation in these patients [110].

Nevertheless, disease recurrence remains a major challenge, occurring in up to 60–70% of cases, most commonly as slowly progressive pulmonary metastases [5,109,110]. However, the pattern and biology of recurrence post-transplantation appear to be more indolent than in non-transplanted cohorts, and some patients may benefit from repeat resection or systemic therapy [5,108,109,110]. Novel biomarkers, particularly circulating tumor DNA (ctDNA), are being explored to enhance candidate selection and enable earlier detection of recurrence, with several prospective studies currently underway [41,42].

As evidence accumulates from trials such as SECA-III, COLT, RAPID-Padova, and LIVER-T(W)O-HEAL, the role of LT for unresectable CRLM is being increasingly defined [110]. Consensus guidelines have begun to incorporate molecular and anatomical criteria—such as exclusion of *BRAF*-mutant and MSI-high tumors—to optimize patient selection. MELD exception frameworks are also being developed to facilitate equitable access to transplantation for oncological indications [109,110].

Despite these encouraging results, several important limitations must be considered. Organ scarcity remains a major barrier to the widespread adoption of LT for CRLM, particularly in healthcare systems with prolonged waiting times and competing indications for transplantation [108,109,110]. The allocation of deceased donor grafts to patients with metastatic disease remains ethically controversial, as these organs may compete with established indications such as end-stage liver disease, hepatocellular carcinoma, or acute liver failure. Consequently, expanding transplant eligibility for CRLM requires careful balancing of individual patient benefit against broader principles of equity, distributive justice, and optimal utilization of a scarce public resource [108,109,110].

Additional challenges relate to the long-term consequences of transplantation itself. Lifelong immunosuppressive therapy increases the risk of infectious complications, metabolic disorders, renal dysfunction, and secondary malignancies, all of which may influence long-term outcomes and quality of life. Furthermore, although liver transplantation may prove cost-effective in carefully selected patients who achieve prolonged survival, robust health-economic evaluations remain limited and should be considered alongside clinical outcomes when assessing the broader applicability of this strategy [5,108,109,110].

Access to liver transplantation also varies considerably across healthcare systems owing to differences in donor availability, organ allocation policies, transplant infrastructure, and reimbursement models. Consequently, the favorable outcomes reported by high-volume transplant centers may not be readily reproducible in regions with more limited resources, highlighting the importance of interpreting current evidence within the context of local healthcare systems [108,109,110].

Liver transplantation is emerging as a potentially valuable treatment option for highly selected patients with unresectable CRLM; however, its role continues to evolve as evidence accumulates from ongoing prospective studies [5,108,109,110]. Broader implementation will depend not only on continued refinement of patient selection and recurrence surveillance strategies but also on addressing unresolved ethical, economic, and resource-allocation challenges. Until additional multicenter prospective data become available, LT should remain confined to carefully selected patients managed within experienced multidisciplinary transplant programs [5,6,108,109,110].

## 5. Conclusions

The management of unresectable colorectal liver metastases has evolved toward an increasingly individualized treatment paradigm in which careful patient selection remains the cornerstone of optimal care. Current evidence strongly supports the use of modern systemic therapy, including conversion chemotherapy and biomarker-guided targeted treatments, to increase resectability rates and improve survival outcomes. In selected patients with limited hepatic disease, local ablative techniques have also demonstrated durable local control and may serve as effective alternatives or complements to surgical resection.

In contrast, the role of several other locoregional strategies remains less clearly defined. Although transarterial therapies, including TACE and radioembolization, may provide disease control in selected settings, their optimal indications, sequencing, and impact on long-term survival require further prospective validation. Similarly, liver transplantation has emerged as a promising option for highly selected patients with liver-confined disease, but broader adoption will depend on ongoing studies evaluating patient selection criteria, oncological outcomes, and resource allocation.

Translating these modalities into practice requires an explicit sequencing strategy rather than parallel consideration. In a patient with liver-only unresectable disease and adequate fitness, first-line therapy is conversion chemotherapy selected by tumour biology, most often a doublet or, in fit patients with right-sided or *RAS/BRAF*-mutant tumours, FOLFOXIRI–bevacizumab, with centrally reviewed radiological reassessment approximately every two months serving as the principal decision node. Response governs the subsequent step: patients who reach technical resectability proceed to metastasectomy, whereas those limited by an insufficient future liver remnant are directed to volume-modulating strategies (portal vein embolization, two-stage hepatectomy, or ALPPS). In selected patients who respond but remain unresectable, liver-directed therapies may be integrated rather than substituted, hepatic arterial infusion (and, less established, radioembolization) to intensify hepatic control with conversion intent, and stereotactic body radiotherapy or thermal ablation to consolidate oligometastatic residual disease, although the supporting evidence is weaker (largely phase II and retrospective) than for systemic conversion or for ablation of small lesions, where level I data exist. For the narrow subgroup with liver-confined, biologically favourable disease showing sustained response yet durable unresectability, transplantation is increasingly considered, with response to prior systemic therapy and tumour biology, rather than anatomy alone, determining candidacy. When no strategy achieves resectability, treatment reverts to a palliative continuum of care. It is this sequencing, anchored in repeated multidisciplinary reassessment and weighted by the strength of evidence behind each modality, rather than the modalities in isolation, that defines contemporary management.

Future research should focus on identifying robust biomarkers capable of refining treatment selection, integrating circulating tumor DNA into clinical decision-making, and defining the optimal sequencing of systemic, surgical, and locoregional therapies. Additional prospective trials are needed to clarify which patients derive the greatest benefit from transplantation and other emerging treatment strategies. Ultimately, the integration of biological, radiological, and clinical data will be essential to advance precision medicine and improve long-term outcomes in patients with unresectable colorectal liver metastases.

Importantly, much of the evidence supporting locoregional therapies and liver transplantation originates from highly selected patient populations, single-center experiences, or non-randomized studies. Therefore, careful interpretation is warranted, and further prospective randomized data are needed before broader adoption of several of these strategies can be recommended.

## Data Availability

No new data were created or analyzed in this study. Data sharing is not applicable to this article.
